# Contribution of macro- and micronutrients intake to gastrointestinal cancer mortality in the ONCONUT cohort: Classical vs. modern approaches

**DOI:** 10.3389/fnut.2023.1066749

**Published:** 2023-01-23

**Authors:** Rossella Donghia, Vito Guerra, Pasqua Letizia Pesole, Marina Liso

**Affiliations:** National Institute of Gastroenterology, IRCCS “S. de Bellis,” Research Hospital, Bari, Italy

**Keywords:** gastrointestinal cancer, machine learning, biostatistics, cancers, nutrition

## Abstract

The aim of this study was to evaluate the contribution of macro- and micronutrients intake to mortality in patients with gastrointestinal cancer, comparing the classical statistical approaches with a new generation algorithm. In 1992, the ONCONUT project was started with the aim of evaluating the relationship between diet and cancer development in a Southern Italian elderly population. Patients who died of specific death causes (ICD-10 from 150.0 to 159.9) were included in the study (*n* = 3,505) and survival analysis was applied. This cohort was used to test the performance of different techniques, namely Cox proportional-hazards model, random survival forest (RSF), Survival Support Vector Machine (SSVM), and C-index, applied to quantify the performance. Lastly, the new prediction mode, denominated Shapley Additive Explanation (SHAP), was adopted. RSF had the best performance (0.7653711 and 0.7725246, for macro- and micronutrients, respectively), while SSVM had the worst C-index (0.5667753 and 0.545222). SHAP was helpful to understand the role of single patient features on mortality. Using SHAP together with RSF and classical CPH was most helpful, and shows promise for future clinical applications.

## Introduction

**FIGURE 1:**
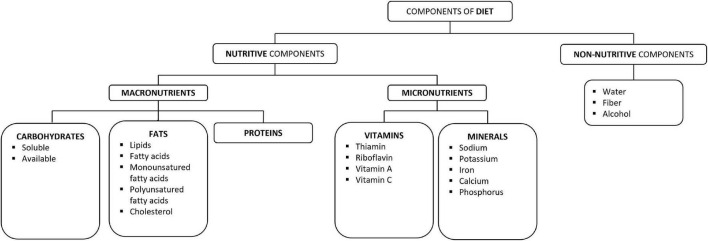
Component of diet.

Cancer is one of the deadliest diseases in the world. Biologically, it includes a collection of diseases in which the cells of the body start dividing uncontrollably without stopping. Normally, human cells grow and duplicate according to physiological needs and their number is controlled by the apoptotic process. In pathological conditions such as cancer, however, cells grow abnormally, old and damaged cells are not eliminated, and new cells duplicate uncontrollably. Malignant tumors invade surrounding tissues and compete with normal cells for nutrients. Additionally, these types of tumors can metastasize to new sites, often distant from the primary tumor site, through the blood or lymphatic system (1, 2). According to the American Cancer Society annual report, 1,918,030 new cases of cancer were recorded in 2022 resulting in 609,360 deaths (3). The most common site of cancer development is the digestive system, and 343,040 new cases were discovered in that same year. The 5-year survival rate for all cancers has grown dramatically since the early 1960s, reaching nearly double, from 39 to 70% among white individuals and triple, from 27 to 63% among black patients. In particular, for gastrointestinal tumors, interest is now focused not only on the tumor growth rate but also on the cellular microenvironment, which is responsible for tumor initiation, progression and metastasis (4, 5). One of the many causes of this dysregulation is certainly an incorrect diet (Figure 1), lifestyle, and genetic background (6) which leads to an ever increasing rate of obesity and continues to remain the “elephant in the room” (7, 8).

Dietary intakes of macro- and micronutrients have been implicated in the etiology of chronic disease as gastrointestinal cancer (9, 10). Several studies have shown that high dietary intakes of macronutrients (as fat, protein and carbohydrate) may have a positive association with the risk of developing of cancer (11). Additionally, fruits and vegetables as sources of dietary fiber, folate, vitamin C, and a lot of phenols, and flavonoids could protect, because are involved on trapping free radicals and reactive oxygen molecules at the cellular level [induced lipid peroxidation has been implicated in malignant transformation (12)], thus acting as a protective mechanism against the oxidative damage during digestion process (13, 14). However, micronutrients with a significantly reduced intake also are involved in other kind of cancer, as the breast cancer risk in obese women (15).

The obesity, which is a slow and progressive condition, is mainly based on an ever increasing fatty acid intake vs. low amounts of fiber, vitamins and minerals, widely present in fruit and vegetables (16, 17). For example nuts contain magnesium, unsaturated fatty acids, potassium, fiber, and vitamin E (18), while dairy products and eggs (19) contain a well-balanced composition of micronutrients that could help for the prevention of hypertension (20). However, also consumption of particular type of vegetable food as N. sativa could reduce oxidative stress and inflammation with various mechanisms including a reduction of lipid peroxidation via its antioxidant properties; agonist of PPAR-γ in adipose tissue; activation of AMPK, increased antioxidants inhibition of NF-κB pathway (21, 22).

The obesity rate associated with the development of gastrointestinal cancer (GC) is estimated to rise by about 18% overall by 2025, affecting 18% of men and 21% of women. Studies have shown that weight disorders such as overweight and obesity are usually associated also with sleep problems, and some studies have shown that adherence to DASH diet has helped to correct this trend (23). Furthermore, these disorders could be associated with the Non-alcoholic Fatty Liver Disease (NAFLD) dyslipidemia, and insulin resistance (24).

One of the many opportunities available to study this increase, as well as the classical clinical approaches, is to apply machine learning (ML) as a basis on which to predict cancer survival. A huge amount of research has been conducted across a wide range of statistical approaches. Classical survival analysis is a well-established methodology for estimating a patient’s probability of survival. Although this is now validated, research is also focused on identifying new methods for prediction and calculating their accuracy. In this work we aim to evaluate survival of GC patients using the classical statistical method, Cox Proportional Hazards (CPH) and compare it with new generation algorithms such as Random Survival Forest (RSF), and Survival Support Vector Machine (SSVM), together with a new approach to quantify the power of parameters’ prediction on GC, denominated Shapley Additive Explanation (SHAP) (Table 1).

**TABLE 1:** General view of machine learning techniques.

Methods	Assumptions	Advantages	Disadvantages
CPH	Proportion hazard ratios are constant over time	Distribution knowledge of survival times not required	Not easy to interpret
RSF	Adapts tree structured for survival analysis	On single survival tree	Not easy to interpret for categorical variables
SSVM	Adapts support vector regression for survival analysis	Works well for unstructured data and it is good for scaling	Not easy to interpret
SHAP	Features independence	The prediction is fairly distributed among the feature values	It is possible to create intentionally misleading interpretations

The use of ML approaches proved to enhance the classical statistical methods, improving cancer diagnosis, prognosis, detection and prediction (25), thus providing researchers with powerful tools. The use of classical approaches, ML methods and SHAP is helpful to solve the problem of the ML algorithm, because it is based on the “black-box” concept (26).

### Models

#### Cox proportional hazard

Survival analysis is an integral part of inferential statistics, in which a time variable describes when an event will occur (27). In fact, if we consider studies recruiting patients, it must be possible to describe each of them based on two values, the duration of the condition (t) and the status (alive/event). The time between the initial moment and the terminal event (not necessarily death) is represented by a random variable *T* (*T* ≥ 0), defined as the “survival time.” Mathematically, survival analysis is linked to conditional probability, that represents whether a patient will survive after a certain time t from his entry into the study, and is therefore conditioned by the fact that the patient survived the previous days. Thus, we also define survival analysis as cumulative probability or cumulative survival. Supposing that this random variable *T* confers a certain probability of surviving, we define F(*t*) as the probability function with a certain density. This distribution function of *T* is of the type:


$$F\left(t\right)=P\left(T<t\right)=\int_{0}^{t}f\left(u\right)du$$

This represents the probability that the survival time will be less than the value of *t*. The survival function S(*t*) is defined as the probability that the survival time will be greater than or equal to *t*, according to this following complementary equation with distribution function:


S(t)=P(T≥t)=1-F(t)

The survival function can be used to represent the probability that an individual will survive beyond the initial time for a certain time *t*. Survival analysis methods are classified as non-parametric, semi-parametric and parametric, based on the assumptions made on the distribution of *T*.

The CPH model is a form of multivariate survival analysis that can control other factors. The dependent variable is time to event (or survival time), which can be death or a clinical event (e.g., myocardial infarction) and is generally used to study the association between survival time and potential predictor variables (28). Specifically, the goal of this model is to evaluate the effect of multiple factors on survival, as well as to examine the occurrence rate of a given event at a particular moment of time. This rate is also called the “Hazard.” The predictor variables are called covariates. The Cox model is defined with the Hazard function which can be defined as follows:


$$h\,\left(t\right)=h_{0}\,\left(t\right)\times \text{exp}\,\left(b_{1}\,x_{1}+b_{2}\,x_{2}+\cdots +b_{p}\,x_{p}\right)$$

where *t* represents the survival time, while h_0_ is the basis of the hazard, exp (b_1_) is the Hazard Ratio. In this way, values equal to 1 can be taken to mean the absence of risk, values > 1 an increased risk, while when < 1 the protection from the risk itself. The applicability of this model must be preceded by an analysis to demonstrate the assumption of proportionality, or to demonstrate what effect each covariate has on the constant hazard function for each time. When this assumption is not proven, then a stratified Cox model should be employed. Generally this condition is rarely unproven, so the Cox model is widely used. It can also be shown graphically by means of a graph of the Schoenfeld residuals, which must be non-random to demonstrate the proportionality of the previously fitted model (29). As in the case of the regression model, the Cox model can also be performed “net” of certain covariates, in order to purify the effect of the association under study.

#### Survival random forest

Random Survival Forest is an adaptation of classical Random Forest (RF) because it includes survival parameters such as status and time. In general, these techniques are classified as an ensemble tree method, i.e., based on classification trees.

An RSF is computed by an ensemble of binary decision trees which can be used to select the most important variables linked with time to event in terms of mortality. Bootstrapping and random node splitting are applied to obtain an ensemble of independent decision trees.

Variable predictiveness can be assessed using variable importance measures for both single and grouped variables.

In a selection process the error rate is approximated by the Out-Of-Bag error (OOB) during the training process. In each tree of the random forest, the OOB error is calculated based on predictions of observations (30).

The variable selection is implemented using the minimal depth of a respective variable, determined in each decision tree of a RSF as the distance from the root node to the closest node (31).

#### Survival support vector machines

The SSVM methodology was developed by Cortes and Vapnik (31) and Kiang et al. (32). These models are based on discriminating two classes of observations by a linear decision surface (defined as the hyperplane) maximizing the distance between the hyperplane and single observations. If the classes are not separable by a linear surface, a non-linear transformation can be obtained through mapping the data on a different dimension space (feature space). By using a kernel function, it is possible to construct the separating hyperplane without explicitly carrying the map into feature space (33).

These models are a powerful tool to analyze this type of data because of their performance in analyzing sparse data, i.e., data with many or more predictors than observations. SSVMs have been widely applied to analyze binary outcomes or for datasets with a survival outcome.

#### Shapley Additive Explanations (SHAP)

This was proposed for the first time by Molan (34). It is a particular type of model based on tree features importance, but with greater accuracy and consistency properties. A Sharpley value is defined as the average marginal contribution of feature values across all possible feature coalitions. Each value can be interpreted as the difference between the actual prediction and the average prediction on the whole dataset (35).

In general, the SHAP values test each combination of predictors to assess the effect of each single predictor, based on the game theory and conditional assumptions (36).

#### Concordance index

This index measures how well models predict time to death of patients. It is easy to interpret because a value of c = 0.5 represents the average performance, i.e., no predictive discrimination, while a model with c > 0.5, predicts well and shows a good capability to distinguish patients with events, whereas c < 0.5 is defined as the worst model (37).

## Materials and methods

### Ethical considerations

The studies were conducted in accordance with the principles of the Declaration of Helsinki and approved by the local Ethics Committee. All enrolled subjects provided written informed consent.

### Participants

In 1992, the ONCONUT prospective cohort was started with the goal of evaluating the relationship between diet and cancer development in a Southern Italian elderly population (*n* = 35,000). The study was sponsored by the Italian National Institute of Health and carried out by the Epidemiology and Biostatistics Laboratory of the National Institute of Gastroenterology “Saverio De Bellis,” Research Hospital, Castellana Grotte (Bari), Italy. From April 1992 to July 1993, patients referred to the Clinical Pathology Laboratories of the three Unità Sanitaria Locale (USL) Bari 16 areas (Municipalities of Monopoli and Polignano a Mare (Bari), Italy), BA 17 (Municipalities of Gioia del Colle and Santeramo in Colle (Bari), Italy) and Bari 18 (Municipalities of Castellana Grotte, Turi, Putignano, Noci, Alberobello and Locorotondo (Bari), Italy) were estimated to amount to 11,622, but only 5,632 subjects (48.46%) (ONCONUT 1) completed about 90% of the semi-quantitative food frequency questionnaire (FFQ). After 5 years, 4,563 patients returned to the Clinical Pathology Laboratories, and compose the ONCONUT 2. After excluding cases other than those with gastrointestinal disease (other types of cancers), 3,505 (76.81%) presented complete data for survival analysis.

The survival rate with GC (ICD-10, codes from 150.0 to 159.9) during these years 1992–1993 (ONCONUT 1) was considered as the main outcome. Food conversion into nutrients (macro- and micronutrients) and calories was performed using the Italian National Institute of Nutrition Food Composition. Tables were integrated with data from Fidanza (38), using a validated semi-quantitative FFQ administered to participants. The glycemic index (GI) derived from each food (39) was calculated using tables and the glycemic load (GL), as suggested by Foster-Powell et al. (40). The present investigation was conducted following the “Standards for Reporting Diagnostic Accuracy Studies” (STARD) guidelines, and the manuscript was organized following the “Strengthening the Reporting of Observational Studies in Epidemiology—Nutritional Epidemiology” (STROBE-nut) guidelines (41).

### Statistical analysis

Patients characteristics are reported as Mean ± Standard Deviation (M ± SD) for continuous variables, and as frequencies and percentages (%) for categorical variables.

To test the associations between groups (Dead *vs* Alive), Chi-square test or Fisher’s exact test for categorical variables were applied, as necessary, while the Wilcoxon rank sum (Mann-Whitney) test was applied for continuous variables.

For studying the risk on mortality, the Cox model was used. The CPH model was fitted to the data, and the proportional hazard assumption was evaluated by means of Schoenfeld residuals (SRT).

We randomly split the data into the training and testing subgroups for CPH, RSF and SSVM.

The training data included 75% of the sample (*n* = 2,629) while the remaining data, the test data, accounting for 25% (*n* = 876), were used to test the model.

To ensure repeatability in terms of estimation, the same seed was used.

After running these models the C-Index was used to evaluate the performance of the model on the test subset. Finally, to compare differences between C-Index values from the models values, paired Student’s *t*-test was used.

When testing the null hypothesis of no association, the probability level of error, at two-tailed, was 0.05. All statistical computations were made using StataCorp. (42) and RStudio software (“Prairie Trillium” Release).

## Results

We randomly subdivided patients and allocated 75% to the training set, and the remaining 25% to the test set.

The demographic and food habits are reported in Table 2 for the total cohort. Mean age was 65.01 ± 8.76 years and 38.26% of patients were male.

**TABLE 2:** Baseline characteristics of patients and macro- and micronutrients intake.

Parameters*	M ± SD or %
Age (years)	65.01 ± 8.76
Gender (M) (%)	1,341 (38.26)
**Educational level (%)**	
None	956 (20.45)
Primary school diploma	2,045 (58.78)
Middle school diploma	324 (9.31)
Diploma	127 (3.65)
University degree	27 (0.78)
Smoking habits (yes) (%)	377 (10.90)
**Marital status (%)**	
Single	168 (4.90)
Married or cohabiting	2,622 (76.51)
Separated or divorced	32 (0.93)
Widower	605 (17.65)
BMI (kg/cm^2^)	26.53 ± 4.27
Glycemic index	56.15 ± 4.67
Glycemic load	135.83 ± 71.57
Diabetes (yes) (%)	766 (23.00)
Myocardial infarction (yes) (%)	195 (5.99)
**Macronutrients^ψ^**	
Water (g)	1790.75 ± 731.44
Proteins (g)	69.05 ± 29.49
Lipids (g)	76.22 ± 27.94
Available carbohydrates (g)	250.89 ± 121.05
Fatty acids (g)	131.72 ± 74.22
Soluble carbohydrates (g)	101.86 ± 63.87
Total fiber (g)	26.28 ± 13.89
Saturated fatty acids (g)	20.36 ± 9.25
Monounsaturated fatty acids (g)	40.52 ± 15.07
Polyunsaturated fatty acids (g)	8.31 ± 3.21
Cholesterol (mg)	183.55 ± 105.68
Alcohol (mg)	15.41 ± 19.76
**Micronutrients^ψ^**	
Sodium (mg)	1450.37 ± 837.52
Potassium (mg)	3330.30 ± 1658.44
Iron (mg)	11.19 ± 4.92
Calcium (mg)	851.19 ± 469.13
Phosphorus (mg)	1144.59 ± 483.75
Thiamin (mg)	0.78 ± 0.35
Riboflavin (mg)	1.41 ± 0.62
Vitamin A (μg)	1146.33 ± 942.73
Vitamin C (mg)	170.74 ± 122.68

ONCONUT study (n = 3,505), total cohort. *As mean and standard deviation (M ± SD) for continuous variables and percentage (%) for categorical variables.

BMI, body mass index.

^ψ^Calculated on quantity of daily consumption.

Table 3 shows the difference between alive and dead GC patients. Patients who were older, male, separated, divorced or widower, and with lower education levels were more represented than the others, likewise patients with diabetes and myocardial infarction (*p* < 0.001 respectively). Lower intakes of both macro- and micronutrients were confirmed in dead patients, where all comparisons resulted statistically significant (*p* < 0.05).

**TABLE 3:** Comparison between patients with and without mortality event in the total cohort.

Parameters*	Status		
	**Alive (*n* = 2,847)**	**Died (*n* = 658)**	***p*** **^§^**
Age (years)	63.49 ± 8.05	71.57 ± 8.67	<0.0001
Gender (M) (%)	1,001 (35.16)	340 (51.67)	<0.001^∧^
Educational level (%)			0.01^∧^
None	744 (26.32)	212 (32.52)	
Primary school diploma	1,676 (59.29)	369 (56.60)	
Middle school diploma	274 (9.69)	50 (7.67)	
Diploma	111 (3.93)	16 (2.45)	
University degree	22 (0.78)	5 (0.77)	
Smoking habit (yes) (%)	310 (11.03)	67 (10.34)	0.61^∧^
Marital status (%)			<0.001^∧^
Single	137 (4.91)	31 (4.87)	
Married or cohabiting	2,192 (78.57)	430 (67.50)	
Separated or divorced	26 (0.93)	6 (0.94)	
Widower	435 (15.59)	170 (26.69)	
BMI (kg/cm^2^)	26.65 ± 4.29	26.02 ± 4.17	0.002
Glycemic index	56.21 ± 4.63	55.90 ± 4.83	0.18
Glycemic load	138.06 ± 71.80	126.17 ± 69.80	<0.0001
Diabetes (yes) (%)	563 (20.83)	203 (32.32)	<0.001^∧^
Myocardial infarction (yes) (%)	120 (4.54)	75 (12.20)	<0.001^∧^
**Macronutrients^ψ^**			
Water (g)	1806.86 ± 725.56	1721.02 ± 752.93	0.0002
Proteins (g)	69.98 ± 30.08	65.00 ± 26.47	0.0001
Lipids (g)	77.07 ± 28.10	72.56 ± 26.97	<0.0001
Available carbohydrates (g)	254.54 ± 121.36	235.10 ± 118.49	<0.0001
Fatty acids (g)	133.68 ± 74.60	123.24 ± 71.96	0.0004
Soluble carbohydrates (g)	103.25 ± 63.80	95.86 ± 63.87	0.0002
Total fiber (g)	26.63 ± 13.80	24.76 ± 14.15	<0.0001
Saturated fatty acids (g)	20.62 ± 9.46	19.26 ± 8.24	0.0005
Monounsaturated fatty acids (g)	40.84 ± 14.92	39.14 ± 15.63	0.0006
Polyunsaturated fatty acids (g)	8.41 ± 3.22	7.88 ± 3.15	<0.0001
Cholesterol (mg)	186.21 ± 107.93	172.04 ± 94.55	0.007
Alcohol (mg)	15.55 ± 19.80	14.79 ± 19.55	0.26
**Micronutrients^ψ^**			
Sodium (mg)	1480.34 ± 842.00	1320.69 ± 805.72	<0.0001
Potassium (mg)	3378.20 ± 1644.73	3123.04 ± 1702.33	<0.0001
Iron (mg)	11.35 ± 4.88	10.51 ± 5.05	<0.0001
Calcium (mg)	860.72 ± 472.95	810.57 ± 450.34	0.003
Phosphorus (mg)	1157.39 ± 487.70	1089.20 ± 462.54	0.0004
Thiamin (mg)	0.80 ± 0.35	0.73 ± 0.34	<0.0001
Riboflavin (mg)	1.42 ± 0.62	1.35 ± 0.59	0.002
Vitamin A (μg)	1150.60 ± 914.78	1127.83 ± 1055.71	0.003
Vitamin C (mg)	173.76 ± 121.13	157.66 ± 128.47	<0.0001

*As mean and standard deviation (M ± SD) for continuous variables and percentage (%) for categorical variables.

BMI, body mass index.

^§^ Wilcoxon rank-sum test (Mann-Whitney), ^∧^Chi-square or Fisher’s test, where necessary.

^ψ^Calculated on quantity of daily consumption.

**FIGURE 2:**
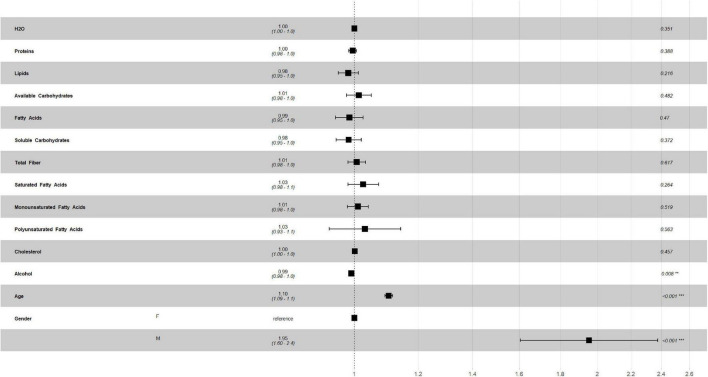
Multivariate Cox regression for macronutrients intake in training set.

**FIGURE 3:**
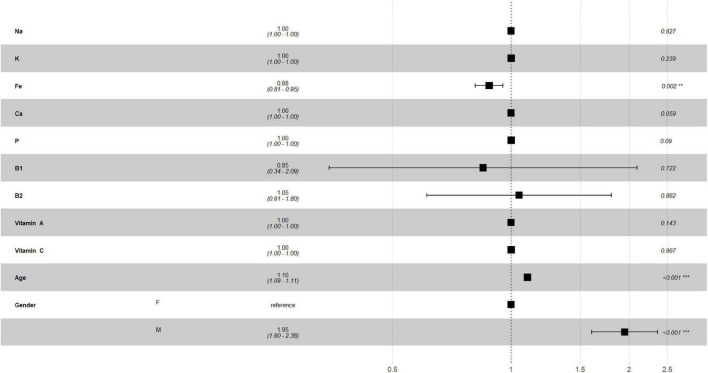
Multivariate Cox regression for micronutrients intake in training set.

Figures 2, 3 describe the traditional Cox model adjusted for age and gender in the training set. For macronutrients (Figure 2) only alcohol intake was associated with risk of mortality, with a low protective role (HR = 0.99, *p* = 0.008, 0.98–1.0, 95% C.I.), while for micronutrients intake (Figure 3) iron intake had a protective role against mortality (HR = 0.88, *p* = 0.002, 0.81–0.95, 95% C.I.).

**FIGURE 4:**
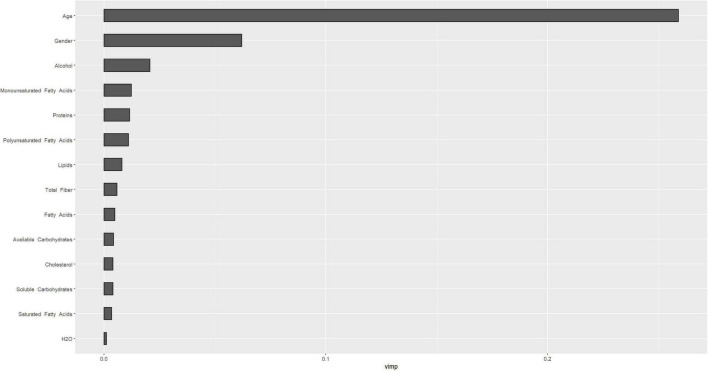
Importance values for macronutrients in training set.

**FIGURE 5:**
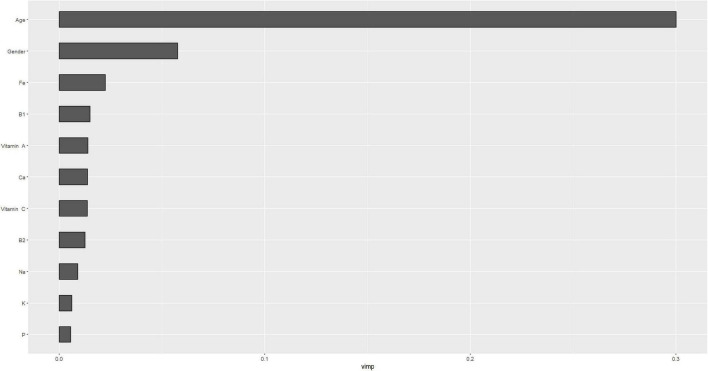
Importance values for micronutrients in training set.

**FIGURE 6:**
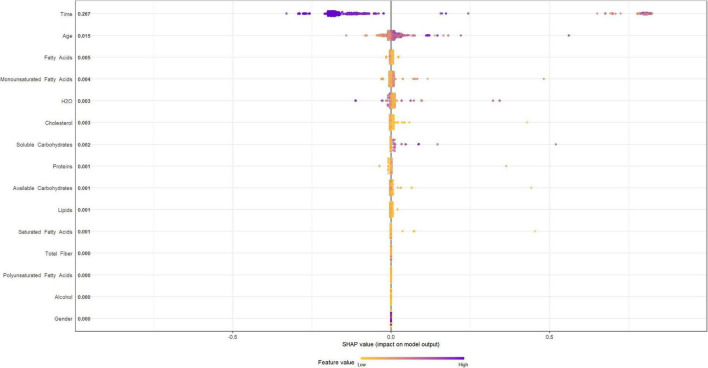
Shapley Additive Explanation summary plot for macronutrients in training set.

**FIGURE 7:**
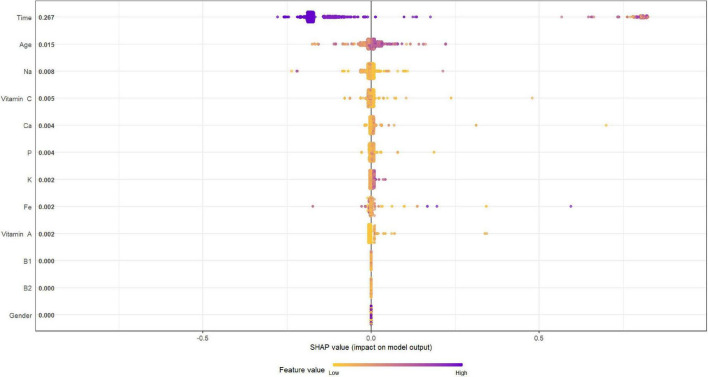
Shapley Additive Explanation summary plot for micronutrients in training set.

Figures 4, 5 describe the importance plot for the RSF for the training set; in both models (Figures 4, 5), alcohol and iron intake, as also the Cox model, had the most important values, contributing to the good prediction of mortality. To identify the features that influenced the prediction, the SHAP summary plot was used (Figures 6, 7). Each point corresponds to a single patient in the testing set. The position on the x-axis, i.e., SHAP value, was the impact of the variable on the model output. Statistically, this represents the logarithm of the mortality risk. Patients with higher SHAP values had a higher risk of death, while lower SHAP values had lower risk. For macro- and micronutrients (Figures 6, 7, respectively) except the time and age variables, all patients had SHAP value near to 0, except for fatty acids (for macronutrients) and Sodium intake (for micronutrients), when more patients had higher SHAP values.

**FIGURE 8:**
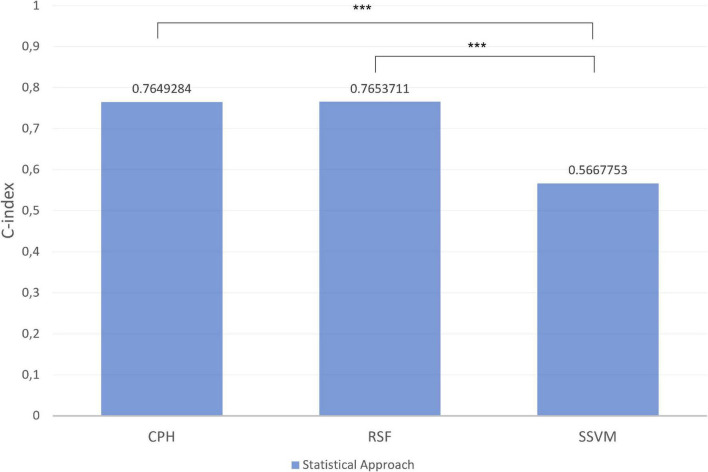
Index in the different models for macronutrients in test set. ***p-value significant. Other comparisons were not significant with *p*-value > 0.05.

**FIGURE 9:**
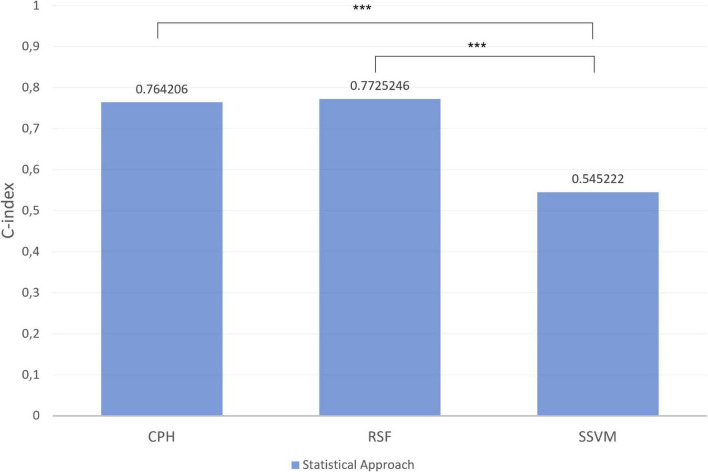
Index in the different models for micronutrients in test set. ***p-value significant. Other comparisons were not significant with *p*-value > 0.05.

Figures 8, 9 depict the quantification of the C-Index in the graph bar. Among macro- and micronutrients CPH and RSF had similar values (0.7649284 and 0.7653711, 0.764206 and 0.7725246, respectively), but the values for SSVM were statistically different for each statistical technique (0.5667753 and 0.545222, respectively, *p* < 0.0001).

## Discussion

In this study we enhanced predictive performances using different survival statistical techniques in an elderly cohort.

Alcohol consumption had a protective role on mortality in our cohort, unlike in the literature (43), probably due to wine consumption rather than spirits. Red wine is a source of polyphenols, such as resveratrol and anthocyanin, with demonstrated antioxidant properties and protective role against cardiovascular diseases (44–46).

In the same way, iron, like alcohol, had a protective role and a high impact on mortality both in the CPH model and RSF (47). Among these methods, RSF demonstrated the best performance followed by CPH, as reported in the literature (48) while the SVM showed the worst performance because generally used for a wide array of applications for its high potential in data transformation and geometric structure (49, 50). The results reveal a promising ML potential for predicting mortality risk in clinical practice. SHAP values on the summary plot were an interesting way to illustrate key features and a good method for describing the power of prediction of outcome. RSF followed by CPH are known to be the best for predicting mortality, while the use of SHAP is still rarely used. The power of SHAP was to allow a description of the single patients variability and also of how individual patients were distributed in terms of SHAP values. The advantage of using SHAP was to overcome the problem of interpreting the classical ML techniques, widely described in the literature regarding the prediction of macro- and micronutrients, and to couple it with other techniques to improve the prediction (51).

The main limitation of this study is the use of an elderly cohort. Mathematically, the use of larger cohorts will surely lead to different results and therefore external validation could be necessary.

Our report can be seen as a first step toward evaluating personalized nutrition in an elderly cohort, and studying the relationship between macro- and micronutrients intake and mortality with different approaches.

Understanding eating habits and therefore the correct intake of macro and micronutrients will be useful for improving the lifestyle of people with obesity who also suffer from other disabling pathologies (18, 24).

This suggests that combining different techniques could be useful in order to personalize the ideal dietary intake. Furthermore, a future follow-up project will be to use these tools to predict changes in food habits in the Southern Italian population. The promise of using ML tools to achieve nutritional phenotyping needs to be explored further in order to set up standard paradigms based on different epidemiological parameters.

Precision nutrition directly addresses metabolic heterogeneity and may serve as a treatment for obesity and other metabolic diseases. Future interventions should examine ways to increase dietary self-monitoring adherence and intervention exposure and consider the development and testing of a specific predictive algorithm (52).

## Conclusion

The results of this study demonstrate that both the CPH and RSV methods performed well, but the combination with SHAP was best. It is difficult to draw conclusions about preferring one model over the other based on this study and the others in the literature, because each model had advantages and disadvantages. In clinical practice none of the mathematical models described could replace another, but all should be used together to make future decisions. In the last years, Artificial intelligence offers major opportunities to improve public health management.

## Data availability statement

The datasets presented in this article are not readily available because IRCCS “S. de Bellis” Hospital property. Requests to access the datasets should be directed to corresponding author.

## Ethics statement

The studies involving human participants were reviewed and approved by the National Institute of Gastroenterology “S. de Bellis,” Research Hospital, Castellana Grotte (BA), Italy. The patients/participants provided their written informed consent to participate in this study.

## Author contributions

RD and ML: conceptualization and writing—original draft preparation. RD: methodology, software, formal analysis, investigation, and data curation. RD, ML, and PP: writing—review and editing. LP: supervision. VG: methodology. All authors have read and agreed to the published version of the manuscript.

## References

[1] Aunan J Cho W Søreide K. The biology of aging and cancer: a brief overview of shared and divergent molecular hallmarks. *Aging Dis.* (2017) 8:628–42. 10.14336/AD.2017.0103 28966806 PMC5614326

[2] Marongiu F Serra M Laconi E. Development versus evolution in cancer biology. *Trends Cancer.* (2018) 4:342–8. 29709258 10.1016/j.trecan.2018.03.007

[3] Siegel R Miller K Fuchs H Jemal A. Cancer statistics, 2022. *CA Cancer J Clin.* (2022) 72:7–33. 35020204 10.3322/caac.21708

[4] Quante M Varga J Wang T Greten F. The gastrointestinal tumor microenvironment. *Gastroenterology.* (2013) 145:63–78. 23583733 10.1053/j.gastro.2013.03.052 PMC4012393

[5] Sukka S The Onconut ^®^ Project Group. The impact of clinical nutrition on cancer therapy: a frequently underestimated perspective. A complementary approach to cancer patients. *Med J Nutr Metab.* (2012) 5:75–9. 10.1007/s12349-012-0105-z 22962628 PMC3432785

[6] Movahed S Tabrizi F Pahlavani N Toussi M Motlah A Eslami S Comprehensive assessment of nutritional status and nutritional-related complications in newly diagnosed esophageal cancer patients: a cross-sectional study. *Clin Nutr.* (2021) 40:4449–55. 33509666 10.1016/j.clnu.2021.01.003

[7] Walsh D Szafranski M Aktas A Kadakia K. Malnutrition in cancer care: time to address the elephant in the room. *J. Oncol. Pract.* (2019) 15:357–9. 10.1200/JOP.19.00165 31188710

[8] Arensberg M Richards J Benjamin J Kerr K Hegazi R. Opportunities for quality improvement programs (QIPs) in the nutrition support of patients with cancer. *Healthcare.* (2020) 8:227. 10.3390/healthcare8030227 32722026 PMC7551760

[9] Tayyem R Bawadi H Shehadah I Abu-Mweis S Agraib L Bani-Hani K Macro- and micronutrients consumption and the risk for colorectal cancer among Jordanians. *Nutrients.* (2015) 7:1769–86. 10.3390/nu7031769 25763533 PMC4377880

[10] Harshman M Aldoori W. Diet and colorectal cancer: review of the evidence. *Can Fam Physician.* (2007) 53:1913–20. 18000268 PMC2231486

[11] Sun Z Liu L Wang P Roebothan B Zhano J Cotterchio M Association of total energy intake and macronutient consumption with colorectal cancer risk: results from a large population-based case-control study in newfoundland and labrador and Ontario. *Canda. Nutr J.* (2012) 11:18. 10.1186/1475-2891-11-18 22449145 PMC3378449

[12] Gonzalez R. Free radiclas, oxidative stress and DNA metabolism in human cancer. *Cancer Investig.* (1999) 17:376–7. 10370368 10.3109/07357909909032882

[13] Millen A Subar A Graubard B Peters U Hayes R Weissfeld J PLCO, cancer screening trial project team. Fruit and vegetale intake and prevalene of colorectal adenom in a cancer screening trial. *Am J Clin Nutr.* (2007) 86:1754–64. 10.1093/ajcn/86.5.1754 18065596

[14] Sato Y Tsubono Y Kakaya N Ogawa K Kurashima K Kuriyama S Fruits and vegetable consumption and risk of colorectal cancer in Japan: the miyagi cohort study. *Public Health Nutr.* (2005) 8:309–14. 10.1079/phn2004681 15918928

[15] Song H Jeong A Mai Tran T lee J Kim M Park B. Association between micronutrient intake and breast cancer risk according to body mass index in South Korean Adult women: a cohort study. *Nutrients.* (2022) 14:2644. 10.3390/nu14132644 35807825 PMC9268499

[16] Tong Y Gao H Qi Q Liu X Li J Gao J High fat diet, gut microbiome and gastrointestinal cancer. *Theranostics.* (2021) 11:5889–910. 33897888 10.7150/thno.56157 PMC8058730

[17] Murphy N Jenab M Gunter M. Adiposity and gastrointestinal cancers: epidemiology, mechanisms and future directions. *Nat Rev Gastroenterol Hepatol.* (2018) 15:659–70. 10.1038/s41575-018-0038-1 29970888

[18] Pahlavani N Rostami D Ebrahimi F Azizi-Soleiman F. Nuts effects in chronic disease and relationship between walnuts and satiety: review on the available evidence. *Obes Med.* (2020) 17:1001736.

[19] Kolahdouz-Mohammadi R Malekahmadi M Calyton Z Sadata S Pahlavani N Sikaroudi M Effect of egg consumption on blood pressure: a systematic review and meta-analysis of randomized clinical trials. *Curr Hypertens Rep.* (2020) 22:24. 10.1007/s11906-020-1029-5 PMC7189334 32114646

[20] Mansouri M Pahlavani N Sharifi F Varmaghani M Shokri A Yaghubi H Dairy consumption in relation to hypertension among a large population of university students: the MEPHASOUS Study. *Diabetes Metab Syndr Obes.* (2020) 13:1633–42. 10.2147/DMSO.S248592 32523363 PMC7234968

[21] Hadi V Pahlavani N Malekahmadi M Nattagh-Eshtivani E Navashenaq J Hadi S Nigella sativa in controlling type 2 diabetes, cardiovascular, and rheumatoid arthritis diseases: molecular aspects. *J Res Med Sci.* (2021) 26:20. 10.4103/jrms.JRMS_236_20 34221050 PMC8240544

[22] Nattagh-Eshtivani E Barghchi H Pahlavani N Barati M Amiri Y Fadel A Biological and pharmacological effects and nutritional impact of phytosterols: a comprehensive review. *Phytother Res.* (2022) 36:299–322. 10.1002/ptr.7312 34729825

[23] Pahlavani N Khayyatzadeh S Banazadeh V Bagherniya M Tayefi M Eslami S Adherence to a dietary approach to stop hypertension (DASH)-style in relation to daytime sleepiness. *Nat Sci Sleep.* (2020) 12:325–32. 10.2147/NSS.S246991 32607032 PMC7292369

[24] Shabgah A Norouzi F Hedayati-Moghadam M Soleimani D Pahlavani N Navashenaq J. A comprehensive review of long non-coding RNAs in the pathogenesis and development of non-alcoholic fatty liver disease. *Nutr Metab.* (2021) 18:22. 10.1186/s12986-021-00552-5 33622377 PMC7903707

[25] Kourou K Exarchos T Exarchos K Karamouzis M Fotiadias D. Machine learning applications in cancer progrosis and prediction. *Comput. Struct. Biotechno. J.* (2015) 13:8–17. 10.1016/j.csbj.2014.11.005 PMC4348437 25750696

[26] Athanasiou M Sfrintzeri K Zarkogianni K Thanopoulou AC Nikit KS. An explainable XGBoost-Based approach towards assessing the risk of cardiovascular diasease in patients in patients with type 2 diabetes mellitus. *Proceedings of the 2020 IEE 20th International Conference on Bioinformatics and Bioengineering (BIBE).* Cincinnati, OH: IEEE (2020).

[27] Tibshirani R. Regression shrinkage and selection via the Lasso. *J R Stat Soc.* (1996) 58:267–88.

[28] Dietrich S Floegel A Troll M Kuhn T Rathmann W Peters A Random survival forest in practice: a method for modelling complex metabolomics data in time to event analysis. *Int J Epidemiol.* (2016) 45:1406–20. 10.1093/ije/dyw145 27591264

[29] Cox D. Regression models and life-tables. *J R Stat Soc.* (1972) 34:187–202.

[30] Ishwaran H Kogalur UB Blackstone E Lauer MS. Random survival forests. *Ann Appl Stat.* (2008) 2:841–60.

[31] Cortes C Vapnik V. Support-vector networks. *Mach Learn.* (1995) 20:273–97.

[32] Kiang Y Xie J Liu W Xi S Huang L Huang W Immunomarker support vector machine classifier for prediction of gastric cancer survival and adjuvant chemotherpautic benefit. *Clin Cancer Res.* (2018) 24:5574–84. 10.1158/1078-0432.CCR-18-0848 30042208

[33] Lungberg S Lee S. A unified approach to interpreting model predictions. *Adv Neural Inf Process Syst.* (2017) 20:4765–74.

[34] Molan C. *Interpretable machine learning*. Self Published. (2020).

[35] Lim S Chi S. Xgboost application on bridge management systems for proactive damage estimation. *Adv Eng Inf.* (2019) 41:100922.

[36] Harrell J Lee K Mark D. Multivariable prognostic models: issues in developing models, evaluating assumptions and adequacy, and measuring and reducing errors. *Stat Med.* (1996) 15:361–87. 10.1002/(SICI)1097-0258(19960229)15:4<361::AID-SIM168>3.0.CO;2-4 8668867

[37] Pierucci P Misciagna G Ventura M Inguaggiato R Cisternino A Guerra V Diet and myocardial infarction: a nested case-control study in a cohort of elderly subjects in a Mediterranean area of souther Italy. *Nutr Metab Cardiovasc Dis.* (2012) 22:727–33. 10.1016/j.numecd.2010.12.002 21482083

[38] Fidanza F. *Nutrizione Umana.* Idelson: Napoli (1984).

[39] FAO/WHO. *Carbohydrate and Human Nutrition.* Rome: FAO (1998).

[40] Foster-Powell K Holt S Brand-Miller J. International tables of glycemic index and glycemic load values. *Am J Clin Nutr.* (2002) 76:5–56. 12081815 10.1093/ajcn/76.1.5

[41] Lachat C Hawwash D Ocké M Berg C Forsum E Hörnell A Strengthening the reporting of observational studies in epidemiology – nutritional epidemiology (STROBE-nut): an extension of the STROBE statement. *PLoS Med.* (2016) 13:e1002036. 10.1371/journal.pmed.1002036 PMC4896435 27270749

[42] StataCorp. *Stata Statistical Software: Release 17.* College Station, TX: StataCorp LLC (2021).

[43] Belle V Papantonis I. Principles and practive of explainable machine learning. *Front Big Data.* (2021) 4:688969. 10.3389/fdata.2021.688969 34278297 PMC8281957

[44] Fan S Hu Y You Y Xue W Chai R Zhang X Role of resveratrol in inhibiting pathological cardiac remodeling. *Front Pharmacol.* (2022) 13:924473. 10.3389/fphar.2022.924473 36120366 PMC9475218

[45] Bohara R Tabassum N Singh M Gigli G Ragusa A Leporatti S. Recent overview of resveratrol’s beneficial effects and its nano-delivery systems. *Molecules.* (2022) 27:5154. 10.3390/molecules27165154 PMC9414442 36014390

[46] Herrera-Bravo J Beltrán J Huard N Saavedra K Saavedra N Alvear M Anthocyanins found in pinot noir waste induce target genes related to the Nrf2 signalling in endothelial cells. *Antioxidants.* (2022) 11:1239. 10.3390/antiox11071239 35883728 PMC9311808

[47] Jin M Cai S Guo J Zhu Y Li M Yu Y Alcohol drinking and all cancer mortality: a meta-analysis. *Ann Oncol.* (2013) 24:807–16. 23104725 10.1093/annonc/mds508

[48] Fonseca-Nunes A Jakszyn P Agudo A. Iron and cancer risk – A systematic review and meta-analysis of the epidemiological evidence. *Cancer Epidemiol Biomarkers Prev.* (2014) 23:12–31. 10.1158/1055-9965.EPI-13-0733 24243555

[49] Statnikov A Aliferis C. Are random forests better than support vector machines for microarray-based cancer classification. *AMIA Annu Symp Proc.* (2007) 2007:686–90. 18693924 PMC2655823

[50] Ben-Hur A Horn D Siegelmann H Vapnik V. *Support Vector Clustering.* Brookline, MA: Microtome Publishing (2001).

[51] Kumar I Scheidegger C Venkatasubramanian S Friedler S. Shapley residuals: quantifying the limits of the shapley value for explanations. *Proceedings of the ICML Workshop on Workshop on Human Interpretability in Machine Learning, July 2020.* New York, NY: WHI (2020).

[52] Popp C Hu L Kharmats A Curran M Berube L Wang C Effect of a personalized diet to reduce postprandial glycemic response vs a low-fat diet on weight loss in adults with abnormal glucose metabolism and obesity. *JAMA Netw.* (2022) 5:e2233760. 10.1001/jamanetworkopen.2022.33760 36169954 PMC9520362

